# On translating technology research into industrial applications

**DOI:** 10.1186/2193-1801-3-S1-K1

**Published:** 2014-12-04

**Authors:** Stephen T C Wong

**Affiliations:** Houston Methodist Research Institute and Houston Methodist Hospital, Weill Medical College, Cornell University, Houston, USA

Basic or pure research advances fundamental knowledge about the world and generates new ideas, principles, and theories, which, while not always immediately usable, form the basis of progress and development in different fields and industries, even starting new ones. For example, computers could not exist without research in mathematical logics and solid state physics over a century ago, while the prevalent world wide web and social media owe much to the fundamental breakthroughs in semiconductors and network theory, for which there was no known practical application at the time. Basic research is more speculative and takes a long time, often decades, to be applied in the real world, and occasionally results in unexpected discoveries.

Applied research, on the other hand, aims to translate fundamental scientific discoveries into practical applications and create an impact in practice in a relatively short time. It often represents an incremental improvement to current processes or applications rather than delivering radical breakthroughs or revolutionizing how practitioners deal with a problem. In medicine, the term, translational research is further introduced to emphasize the research that aims to move “from bench to bedside” or form laboratory experiments through clinical trials to point-of-care patient applications. Different from basic research, applied or translational research is necessarily a much more iterative endeavor and requires close interaction between academics and industry.

Today, the rapid advances of scientific discovery, increased complexity of the multidisciplinary and multi-skilled teams, and fast changing market places, all make it more difficult to conduct applied or translational research for industrial applications. Many students graduating from the tertiary educational institutions are well equipped with specialized theories and principles of basic research and technologies, but are rather ill prepared for the science-to-business marketing and team oriented working environments required to achieve large impact of applied technology research into practice.

The motivation of this talk is to share some of the cases I have experienced first-hand by working in applied or translational research during the past three decades in various industries, including computers (HP), telecommunications (AT&T Bell Labs), finance (Charles Schwab), medical imaging and healthcare IT (UCSF, Philips Healthcare, Harvard Medical School, and Houston Methodist Hospital). The presentation will focus less on the details of technologies, but rather more on the influence of the environments and market forces that drive the applications of such technologies. The lecture will be delivered in an informal, interactive format, rather than didactic.

